# Experimental vs. modeled water use in mature Norway spruce (*Picea abies*) exposed to elevated CO_2_

**DOI:** 10.3389/fpls.2012.00229

**Published:** 2012-10-16

**Authors:** Sebastian Leuzinger, Martin K.-F. Bader

**Affiliations:** ^1^School of Applied Sciences, Auckland University of TechnologyAuckland, New Zealand; ^2^Forest Ecology, ETH ZurichZurich, Switzerland; ^3^Institute of Botany, University of BaselBasel, Switzerland; ^4^Centre of Excellence for Climate Change, Woodland and Forest Health, School of Plant Biology, University of Western AustraliaCrawley, WA, Australia; ^5^New Zealand Forest Research Institute (SCION)Rotorua, New Zealand

**Keywords:** dendrometer, DGVM, FACE, leaf water potential, sap flow, stomatal conductance, vegetation modeling

## Abstract

Rising levels of atmospheric CO_2_ have often been reported to reduce plant water use. Such behavior is also predicted by standard equations relating photosynthesis, stomatal conductance, and atmospheric CO_2_ concentration, which form the core of dynamic global vegetation models (DGVMs). Here, we provide first results from a free air CO_2_ enrichment (FACE) experiment with naturally growing, mature (35 m) *Picea abies* (L.) (Norway spruce) and compare them to simulations by the DGVM LPJ-GUESS. We monitored sap flow, stem water deficit, stomatal conductance, leaf water potential, and soil moisture in five 35–40 m tall CO_2_-treated (550 ppm) trees over two seasons. Using LPJ-GUESS, we simulated this experiment using climate data from a nearby weather station. While the model predicted a stable reduction of transpiration of between 9% and 18% (at concentrations of 550–700 ppm atmospheric CO_2_), the combined evidence from various methods characterizing water use in our experimental trees suggest no changes in response to future CO_2_ concentrations. The discrepancy between the modeled and the experimental results may be a scaling issue: while dynamic vegetation models correctly predict leaf-level responses, they may not sufficiently account for the processes involved at the canopy and ecosystem scale, which could offset the first-order stomatal response.

## Introduction

Whether and if so how plants respond to increasing atmospheric CO_2_ is critical for future ecosystem carbon and water cycling and largely depends on the response of the stomata that control both carbon (C) uptake and water loss. Several authors have suggested that water-related effects of elevated CO_2_ (eCO_2_) on stomatal closure might be or become more important than immediate effects on carbon uptake (Morgan et al., 2003; Holtum and Winter, 2010; Hartmann, 2011). One reason for this may be that while stimulated growth and biomass accumulation rarely persist over many years (Körner et al., 2005; Norby et al., 2010; Leuzinger et al., 2011b), but see McCarthy et al. (2010), stomatal responses tend to be sustained (see Holtum and Winter, 2010 for a review). For grassland, there is compelling experimental evidence that stimulated biomass production under eCO_2_ is in fact a consequence of soil water savings resulting from reduced stomatal conductance (Niklaus et al., 1998; Morgan et al., 2004). Such a water-mediated CO_2_-effect is expected to be more pronounced in water-limited ecosystems, although under extremely dry conditions it has not been observed (Housman et al., 2006). The experimental conditions (species composition tested, CO_2_-administration method, laboratory vs. field experiment, ontogenetic stage of test plants) and particularly the experimental duration are fundamental for the interpretation of net water use under eCO_2_ (Norby et al., 1999). For example, initial CO_2_-induced increases in total leaf area per unit land area leaf area index, (LAI) in young, rapidly expanding systems will inevitably lead to increased stand transpiration, irrespective of the leaf-level stomatal response (Uddling et al., 2008; Tricker et al., 2009). Similarly, the long-term response (>10 years) often differs from measurements over only a few years (Körner, 2006). The scaling from first-order stomatal responses to stand water use therefore requires careful consideration of the interactions between the water- and the carbon cycle across temporal and spatial scales.

Overall, there is a striking lack of data from mature forests as most of the evidence for water savings under eCO_2_ still comes from grass- or shrub ecosystems, or from branch bag experiments on mature trees (Roberntz and Stockfors, 1998; Pataki et al., 2000; Morgan et al., 2004). No eCO_2_ experiments on tall trees exist in the tropical forests (Körner, 2009). Worldwide, there are only five experiments testing the effect of future CO_2_ concentrations on entire tree crowns using the free air CO_2_ enrichment (FACE) method (Körner et al., 2005; Norby et al., 2005), excluding whole tree chamber and agricultural experiments (Medhurst et al., 2006; Kimball et al., 2007). Although those five studies were conducted with different species and in a variety of biomes, three of them roughly agree on reduced total stand water use under eCO_2_ of c. 10% (Warren et al., 2011). The remaining two were young, expanding systems where the transpiration response to CO_2_ was dominated by a stimulation of LAI and therefore increased stand water use (Uddling et al., 2008; Tricker et al., 2009). Responses in leaf-level stomatal conductance (*g*_*s*_) to eCO_2_ are less consistent in the five large forest FACE experiments mentioned (−4% to −44%), but approximately match findings from meta-analyses including experiments with woody plants in branch bag and greenhouse experiments (−21% Medlyn et al., 2001, −18% Ainsworth and Rogers, 2007, and no significant response from a much earlier review predominantly on seedlings and saplings, Curtis and Wang, 1998). At the Swiss Canopy Crane (SCC), where the present study was conducted, stomatal conductance was reduced around 10% in six deciduous tree species, and sap flow by 2–22%, resulting in a reduction of yearly stand transpiration of c. 10% (Cech et al., 2003; Keel et al., 2007; Leuzinger and Körner, 2007). Overall, water savings under eCO_2_ tend to decline with the duration of the experiment (Medlyn et al., 2001; Leuzinger and Körner, 2010; Leuzinger et al., 2011b), with increasing woodiness (Ainsworth and Rogers, 2007), with increasing age of the studied plants (Medlyn et al., 2001), and from deciduous to coniferous trees (Ellsworth, 1999; Körner et al., 2007). Because the number of short-term experiments with herbaceous or young trees is disproportionately larger than the number of experiments with mature trees, we can assume that water savings under eCO_2_ tend to be overestimated, particularly because the response is likely to diminish further when scaling up from the individual plant to the landscape level (McNaughton and Jarvis, 1991; Field et al., 1995; Leuzinger and Körner, 2010). A way to circumvent the necessity of scaling up CO_2_-experiments with young, disturbed systems is Δ^13^C analysis of tree rings to infer intrinsic water use efficiency (iWUE, Francey and Farquhar, 1982; Bert et al., 1997). Penuelas et al. (2011) in a recent review report that iWUE has increased by c. 20% over the past 40 years, with slightly larger responses in broad-leaved relative to coniferous trees. On the other hand, in a study on various species of oak, hornbeam and maple, no change in iWUE was reported based on only 2–3 leaf samples during the twentieth century (Miller-Rushing et al., 2009). Changes in iWUE may originate either from changes in the nominator (leaf-level photosynthesis) or the denominator (stomatal conductance), and thus cannot be used to fill in the lack of FACE experiments to estimate changes in stand transpiration under future CO_2_ concentrations.

Experimental estimates of leaf-level and whole tree responses are also key to algorithms and their parameterization in dynamic global vegetation models (DGVMs) and earth system models, and therefore predictions of future vegetation responses and climate feedbacks (Moorcroft, 2006). Stomatal conductance models used in DGVMs predict substantial decreases of *g*_*s*_ with a doubling of atmospheric CO_2_, fundamentally because the substomatal CO_2_ concentration (*C*_*i*_) is held approximately constant (Jarvis, 1976; Leuning, 1995; Haxeltine and Prentice, 1996). It is therefore little surprising that DGVMs predict global water savings by the vegetation of around 10–20% (Luo et al., 2008), which lead to increased runoff of mostly a few percent (e.g., Betts et al., 2007; Boucher et al., 2009; Long et al., 2010), matching estimates based on experimental data (Leuzinger and Körner, 2010). However, it is important to note that few of these model results can be validated due to a lack of data, and net responses largely hinge on the way the leaf-level response is scaled up to the canopy and landscape scale. The aim of the present study is to (1) provide novel data on water relations of fully grown Norway spruce (*Picea abies*) trees under approximately double pre-industrial CO_2_-conditions, and (2) to discuss these findings in context of simulations of the experiment by the DGVM LPJ-GUESS (Smith et al., 2001; Sitch et al., 2003).

## Materials and methods

### Study site

The SCC is located in a mature, mixed deciduous forest 15 km south of Basel, Switzerland (47°28′N, 7°30′E, 550 m a.s.l.). The crane gondola allows access to all tree crowns located within the 30 m radius of the jib. The site has a mean January temperature of 2°C and mean July temperature of 19°C, long-term average annual precipitation amounts to 990 mm, two-thirds of which fall during the growing season. The oldest trees are c. 110 years old and reach heights of 35–40 m. Stand density is 415 trees ha^−1^ (trees >0.1 m breast height diameter), at a stem basal area of 46 m^2^ ha^−1^ and a LAI of c. 5. The soil type is a Rendzic Leptosol (WRB) (Rendzina, FAO; Lithic Rendoll, USDA) with an accessible profile depth of at most 25 cm followed by rocky subsoil blending into the calcareous bedrock at 40–90 cm. The soil texture is a loamy clay (pH 5.8 in the top 10 cm). The species mixture includes deciduous trees (*Fagus sylvatica* L., *Quercus petraea* (Matt.) Liebl., *Carpinus betulus* L., and, less abundant, *Tilia platyphyllos* Scop., *Acer campestre* L., *Prunus avium* L.) as well as conifers (*Picea abies* (L.) Karst., *Larix decidua* Mill., *Pinus sylvestris* L., *Abies alba* Mill.). For more information on the site see (Pepin and Körner, 2002).

### Experimental set-up and free air CO_2_ enrichment

Five Norway spruce (*P. abies*) individuals were selected for CO_2_ enrichment (550 ppm), together with five control trees, only three of which were accessible with the crane gondola. The treatment was initiated on July 30, 2009. To simulate future atmospheric conditions, pure CO_2_ was released through laser-punched irrigation tubes woven into the spruce trees with a central supply pipe running up the stem. Sample lines were connected to two infrared gas analyzers (LI-800, Li-Cor, Lincoln, NE, USA), in order to monitor and automatically adjust the amount of CO_2_ supplied. CO_2_ enrichment was discontinued when daily temperature maxima did not reach 6°C, or when above-canopy incoming radiation was less than 100 μmol m^−2^ s^−1^ (i.e., no night-time CO_2_ enrichment). On average, the target CO_2_ concentration of 550 ppm was achieved well: the mean across all sample lines during times of fumigation reached 563 ppm ± 94 s.e. in 2009 and 617 ppm ± 88 in 2010.

### Continuous measurements and measuring campaigns

We continuously measured sap flow, stem radius changes, microclimate, and soil moisture. One heat dissipation sap flow sensor (TDP-30, Dynamax, Huston, TX, USA) was used per tree (5 treated, 5 controls), inserted directly into the stem after removing loose bits of bark. The sensors were oriented toward north, water-proofed with silicon paste and insulated with styrofoam and reflecting foil. They remained in the same position for both the 2009 and 2010 growing seasons. Stem radius changes (μm) were recorded with high-precision point dendrometers (ZB06, Natkon, Hombrechtikon, Switzerland), and, together with the sap flow signals, logged to two central data loggers (DL2e, Delta-T Devices Ltd., Cambridge, UK) in 10 min intervals (average over 30 s readings). Soil moisture was logged every 6 h at 0–10 cm depth using “ECH_2_O Probes” (EC-10, Decagon Devices Ltd., Pullman, Washington, DC). Eleven sensors were distributed around the treated trees, 18 sensors around control trees, logging onto self-contained data loggers (Em50, Decagon). Measurements took place from day of year 134 to 297 (May 14–October 24) in 2009 and from day of year 134 to 267 (May 14–September 24) in 2010. Microclimate (temperature, relative humidity, incoming radiation, and precipitation) was logged above the canopy in 10-min intervals to a DL2e logger (Delta-T Devices Ltd.). Data gaps resulting from occasional logger failure were filled by interpolation based on climate recordings from a nearby weather station (2 km air-line distance).

On three cloudless days (July 29, August 6 2009, and July 14 2010), we measured daily courses (five measurements per tree pre-dawn to dusk) of leaf water potential and stomatal conductance on the five treated trees and the three controls that were accessible with the crane gondola. Two twigs per tree and time window were cut off with a razor blade, needles, bark, and phloem around the cut were removed, and their leaf water potential was measured subsequently using a pressure chamber (SKPM 1400, Skye Instruments, Powys, UK). Only plant material from the fully light-exposed top crown was selected to ensure comparable conditions. Stomatal conductance was measured on current and previous year's needles, removing the needles on two c. 2 cm wide bands to allow the gas-exchange chamber to close. A portable photosynthesis system (LI-6400 XT, LI-COR Biosciences, Lincoln, NE, USA) with a conifer chamber was used for this purpose. Readings were taken as soon as rates of net photosynthesis and stomatal conductance remained stable (<5 min). All measurements were taken at full sunlight (>1000 μmol m^−2^ s^−1^). Treated trees were measured at their target CO_2_ concentration of 560 ppm, control trees at ambient CO_2_ (390 ppm). Vapor pressure deficit (VPD) inside the cuvette was adjusted to ambient conditions. Stomatal conductance was calculated by multiplying the readings with the total leaf area of the samples (harvested at the end of the experiment). Because there were no systematic differences between current year and previous year needles, all analyzes presented here are based on the average values.

### Model specification and model runs

We used the DGVM LPJ-GUESS, featuring an accurate representation of detailed plant physiological processes (Smith et al., 2001; Sitch et al., 2003), to simulate our experimental results. The model was run in cohort mode using daily climate data from an official Swiss weather station 11 km north of the study site (Basel-Binningen, Tank et al., 2002). Relative humidity, radiation and temperature data correlated well with weather data recorded at the experimental site (R^2^-values equal to 0.86, 0.89, and 0.98 respectively), and specific correction factors were applied to simulate long climate time series at the experimental site as closely as possible. A 700-years spin-up period was allowed in order to equilibrate the various carbon pools with the background climate (data from 1901 to 1930 used repeatedly, CO_2_ concentration at 296 ppm). Thereafter, we considered the period from 1901 to 2110. Atmospheric CO_2_ concentration was altered in four different ways, all based on the actually measured mean values until 2010: (1) step change to 550 ppm in 2010, (2) step change to 700 ppm in 2010, (3) gradual change to 550 ppm until 2057, and (4) gradual change to 700 ppm until 2100. Because we only considered Norway spruce, only the plant functional type (PFT) “needle-leaved evergreen” was allowed to grow, all other PFTs were suppressed. Soil water storage capacity was set to 80 mm, which is the best estimate determined for a nearby site (see Walthert et al., 2004). Only one patch with a size of 1000 m^2^ was computed, hence no competition took place in order to simulate the responses of mature coniferous trees. Because forest fires are extremely rare in this region, fire disturbance was suppressed. No further parameter changes or adjustments were made relative to the default version of LPJ-GUESS (Sitch et al., 2003).

Daily transpiration in LPJ is equal to the lower value out of daily water supply and daily water demand. If water supply < water demand, then the available soil water is transpired up to a maximum rate of 5 mm d^−1^ and thus independent of CO_2_. On the other hand, if water supply > water demand, stomatal conductance decreases with increasing CO_2_ because *C*_*i*_/C_*a*_ (intercellular to ambient CO_2_ concentration) is held constant and photosynthesis is stimulated (Haxeltine and Prentice, 1996). As a result, at a leaf temperature of 20°C, stomatal conductance is reduced c. 35% at 2× pre-industrial CO_2_ concentration. Transpiration is then calculated from potential evapotranspiration, stomatal conductance and two (constant) scaling parameters.

### Data processing and statistical analyses

Stem water deficit was related to the individual's stem radius at the beginning of the experiment and expressed in per mille change from the initial value. The resulting time series were decomposed into radius changes due to changes in stem water storage and a growth component according to the method outlined in (Zweifel et al., 2005). Essentially, this method considers periods between stem radius peaks as stem water deficits (see Figure 1 of Zweifel et al., 2005).

**Figure 1:**
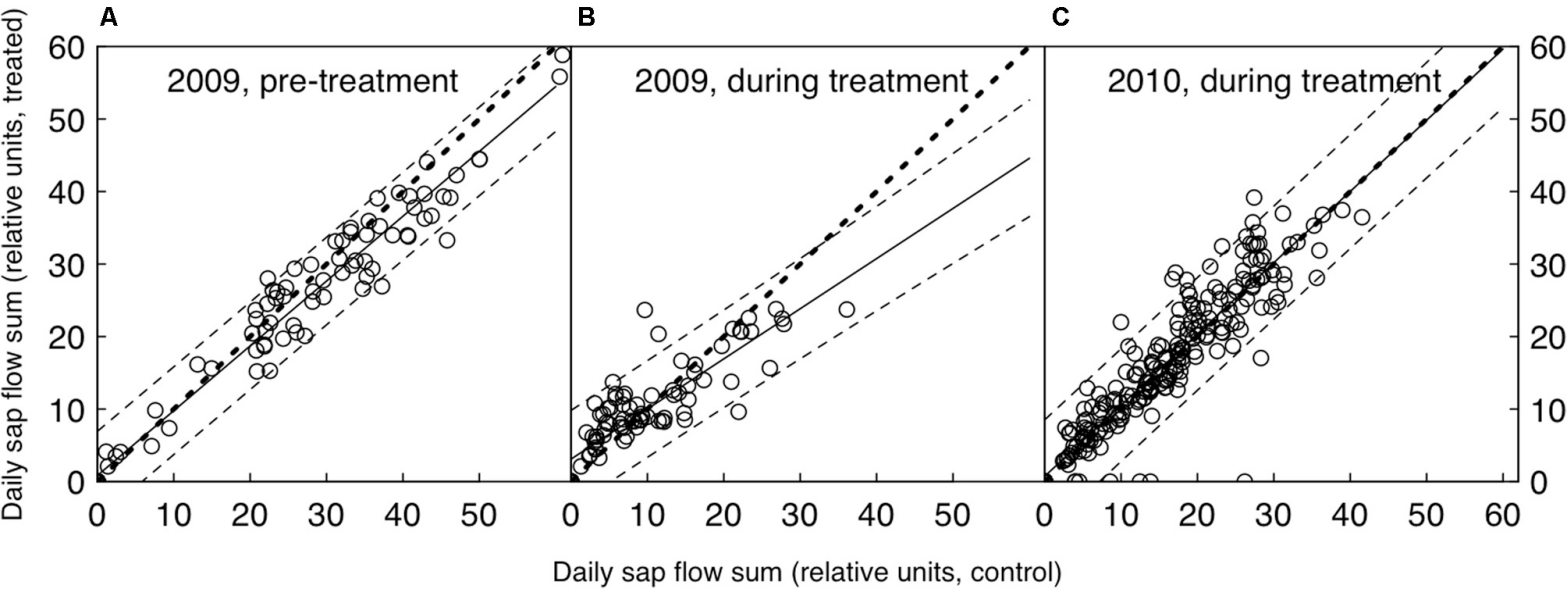
**Daily relative sap flow sums of the control and CO_2_-treated trees, both before (A) and after the CO_2_-treatment was initiated (B and C).** Shown are the medians of each group (*n* = 5). The bold dotted line represents the 1:1 line, the solid line the linear fit (with dashed lines as 95% confidence intervals). The 1:1 line lies within the confidence intervals where data points are available, suggesting that there was no significant difference between treated and control trees in any of the three periods shown.

Millivolt signals of sap flow sensors were processed as follows to achieve most realistic conditions of zero-flow (R. Zweifel, personal communication): the maximum mV values were converted to temperature differences (ΔT) using a constant factor of 25. Then, ΔT maxima between 3 h and 8 h every night were connected with a straight line. This linearly changing baseline (ΔT_max_) was used as the pre-nightly maximum value as in the standard transformation outlined in (Granier, 1985). Because sap flow signals are difficult to interpret as absolute mass flow densities, particularly when exact estimates of the sap wood width and the radial flow distribution are lacking (Leuzinger and Körner, 2007; Leuzinger et al., 2011a), we chose to use only relative sap flow values (Figure 1). Each sap flow time series was therefore standardized to its own pre-treatment maximum (mean of 20 largest values) resulting in time series between 0 and approximately 1, spanning both growing seasons (henceforth called “relative sap flow”). To test treatment-specific differences in daily courses of leaf water potential and stomatal conductance, we used mixed effects models (R package “nlme”) with treatment and time of day as fixed effects and tree as a random effect. Additionally, we used an autoregressive moving average (ARMA) correlation structure to model dependence among observations.

The relationship between stem water deficit (Δ*W*) and soil moisture (*sm*) was modeled individually for the pre-treatment and the two FACE periods (season 2009 and 2010) using a 2-parameter Michaelis-Menten-type hyperbola: Δ*W* = a × *sm*/(b + *sm*), where “a” is the asymptote and “b” the soil moisture level at which stem water deficit reaches half of its asymptotic value (Figure 3).

**Figure 2:**
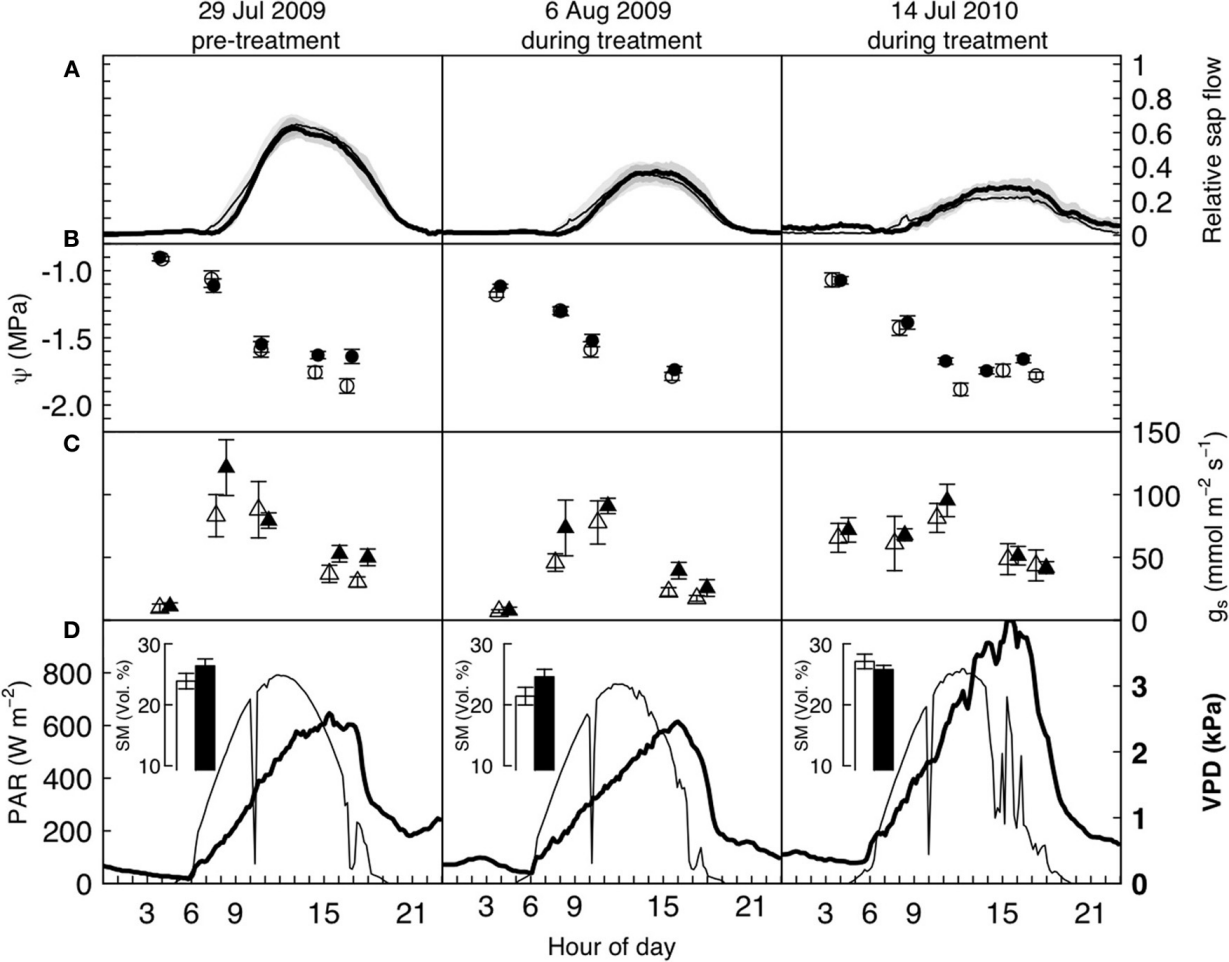
**Diurnal water relations data of mature *P. abies* trees under elevated (bold/filled symbols) and ambient (open symbols) atmospheric CO_2_, before and after the treatment was initiated, on three cloudless summer days in 2009 and 2010.** Panels **(A)** show relative sap flow (each tree standardized to its pre-treatment maximum, *n* = 5), panels **(B)** and **(C)** show mean leaf water potentials and stomatal conductance from pre-dawn to dusk, and **(D)** the photosynthetically active radiation (above the canopy, thin line, left hand side axis) and vapor pressure deficit (at canopy height, bold line, and font, right hand side axis). Shaded areas and bars represent one standard error. None of the differences between control and treated trees is significant on any of the 3 days (see text).

Generally, we tested statistically significant differences between treatments by fitting models with common parameter estimates and varying parameter estimates for each treatment, followed by a comparison of the two models (Figures 2, 4, and 5). The CO_2_-treatment was considered to affect the variable of interest statistically significantly, if the Akaike information criterion (AIC) was significantly lower in the more complex model (i.e., ΔAIC > 2). To determine the envelope curves for the sap flow-VPD relationships, we calculated the 95th percentiles of relative sap flow (*SF*_95_) for every 2 kPa VPDVPD bin and fitted the polynomial model *SF*_95_ = a × *CO*2 + b × VPD + c × VPD^2^ + d × VPD^3^, since all other attempts to fit a non-linear model failed. The factor *CO*_2_ is the CO_2_-treatment with the levels 1 (elevated) and 0 (ambient). The interaction term VPD × *CO*_2_ was not significant and was therefore dropped. All analyzes were carried out using R version 2.13.0 (R Development Core Team, 2011).

## Results

### Water relations of *P. abies* under elevated CO_2_

A comparison of the seasonal relative sap flow sums (median of both treated and control groups) did not show any change when the pre-treatment period was compared to the CO_2_ enrichment period. (Table 1, Figure 1). The pattern did not differ when wet and dry periods were considered separately (both by soil moisture and VPD conditions). Before the treatment was initiated, the trees designated to be treated with eCO_2_ tended to show less transpiration (Wilcoxon rank sum test, *p* = 0.055, Table 1).

**Table 1:** **Comparison of measured and modeled transpiration under control and elevated CO_2_ conditions, estimated from relative sap flow in *P. abies* in the experiment, and from total stand transpiration of evergreen needle-leaved trees in the dynamic global vegetation model LPJ-GUESS**.

	**Control ± s.e.**	**Elevated CO_2_ ± s.e.**	**Difference in %**
**MEASURED**			
**2009** (pre-treatment)	2461 ± 223	2215 ± 162	−10.0% (n.s., *P* = 0.056)
**2009** (during treatment)	1079 ± 175	1058 ± 171	−1.9% (n.s., *P* = 0.095)
**2010** (during treatment)	3435 ± 452	3930 ± 449	+12.6% (n.s., *P* = 0.55)
**MODELED**			
**2010** (step change to 550 ppm in 2010)	0.81 mm/d	0.73 mm/d	−10.0%
**2010** (step change to 700 ppm in 2010)	0.81 mm/d	0.66 mm/d	−18.6%
**2110** (step change to 550 ppm in 2010)	0.73 mm/d	0.66 mm/d	−9.0%
**2110** (step change to 700 ppm in 2010)	0.73 mm/d	0.59 mm/d	−18.4%
**2110** (gradual change to 550 ppm in 2057)	0.73 mm/d	0.66 mm/d	−8.7%
**2110** (gradual change to 700 ppm in 2100)	0.73 mm/d	0.62 mm/d	−15.4%

Seasonal sum of sap flow (March–October, arbitrary units) and transpiration (modeled, mm/d) under control and treatment conditions are shown. The Wilcoxon rank-sum test is based on five replicates (tree individuals) each for control and treated trees.

A more detailed look at the daily courses of sap flow, leaf water potential, and stomatal conductance during bright sunny days before and after the start of the treatment confirmed that water consumption in *P. abies* remained unaffected by CO_2_ enrichment. Daily courses of sap flow were not significantly different between treatments, neither before nor after treatment initiation (daily sums of relative sap flow, Wilcoxon rank-sum test, *n* = 5, *p*-values = 0.90, 0.90, 0.79 for left, center and right panel of Figure 2A). Similarly, leaf water potential and stomatal conductance differed with time of day (*p*-values < 0.0001 for both years and measurements) but not with the treatment: we employed linear mixed effects models with treatment, time and their interaction as fixed factors and tree individuals as random factors. Except for the leaf water potential measurements during the pre-treatment period (left panel of Figure 2B), where the interaction term was significant (*p* = 0.022), the treatment differences were not significantly different during any day (Figures 2B,C, *p*-values > 0.1). In both summers, low soil water availability resulted in low pre-dawn leaf water potentials around −1 MPa. During daytime, high VPD values exceeding 2 kPa caused leaf water potentials to drop to values between −1.5 and −2.0 MPa. Stomatal conductance peaked with light intensity and reached values about 100 mmol m^−2^ s^−1^ and declined in the course of the afternoon to values below 50 mmol m^−2^ s^−1^.

Overall, sap flow was lower on July 14 2010 because of the very high VPD values (maximum of c. 3.5 kPa). All 3 days were cloudless, except for some haziness in the afternoon of July 14 2010. The dent in photoactive radiation (PAR) around 10 am is due to passing shadow from the crane top.

**Figure 3:**
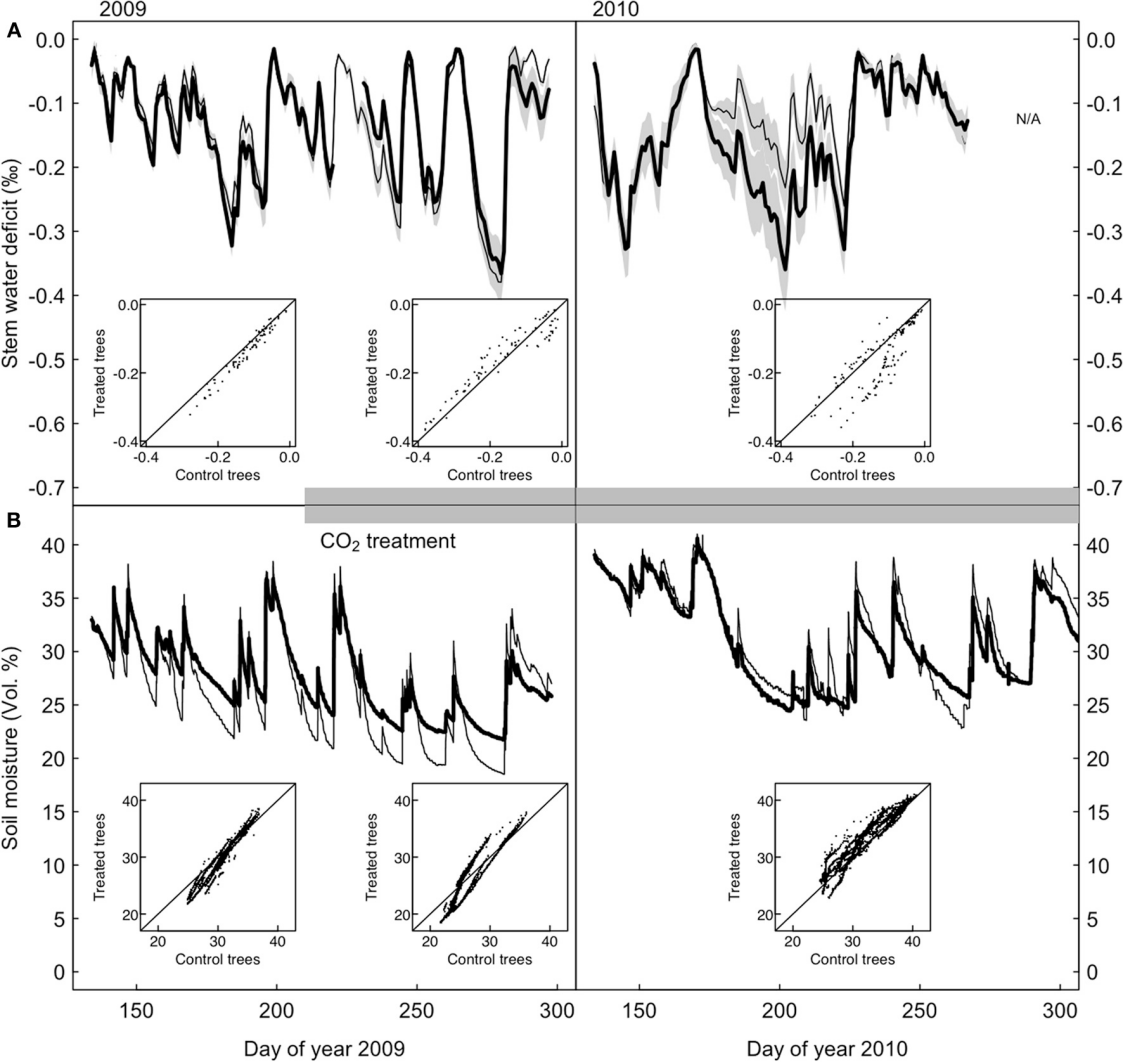
**Stem water deficit in control and CO_2_-treated *P. abies* trees. (A)** Standardized time series of stem water deficit in CO_2_-treated (bold) and control (dashed line) trees, growth-trend corrected (see section “Materials and Methods”). **(B)** Soil water content measured in the 10 cm top soil layer for the control and treated area. The insets show that there are no systematic differences between CO_2_-treated and control trees during any of the three periods.

**Figure 4:**
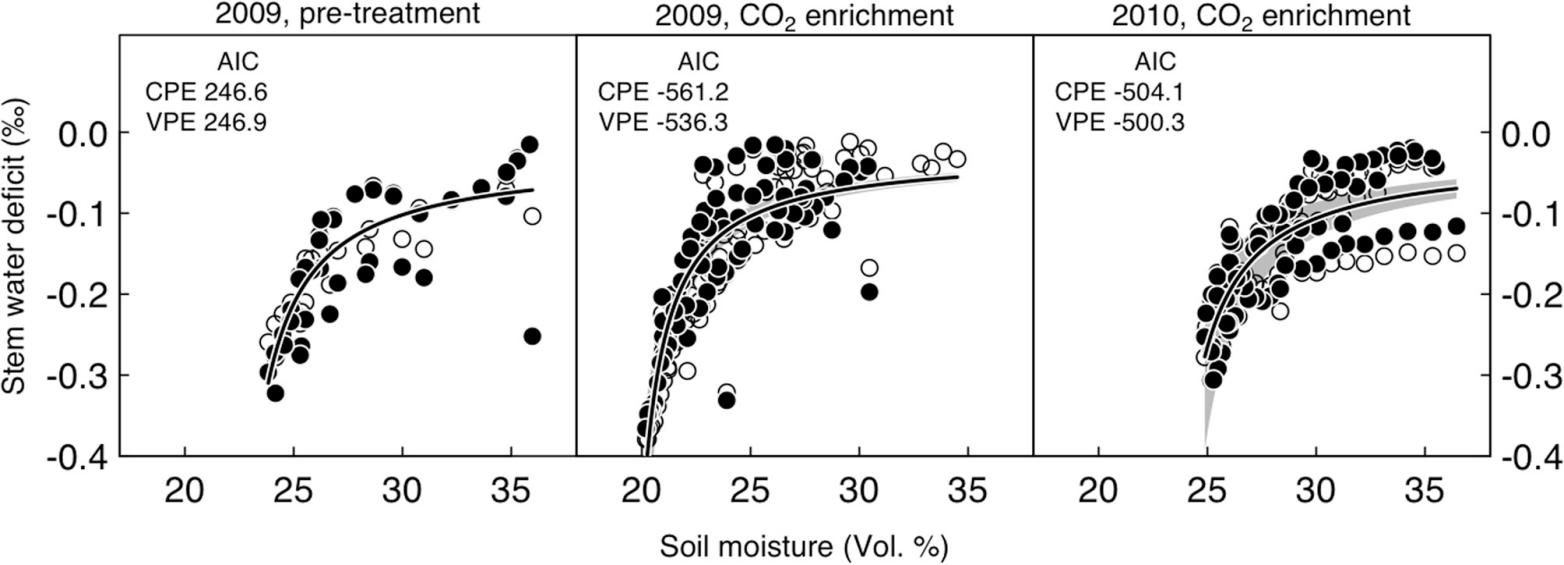
**Relationship between soil moisture (mean across ambient and CO_2_-treated area) and stem water deficit in per mille for *P. abies* under ambient (open symbols) and elevated CO_2_-conditions (filled symbols).** Periods between day of year 175 and 250 were considered. AIC, Akaike Information Criterion; CPE, nonlinear model with common parameter estimates; VPE, nonlinear model with varying parameter estimates for each treatment. Soil moisture explained 77%, 82%, and 65% of the variation in stem water deficit during the pre-treatment period and the 2009 and 2010 CO_2_ enrichment periods, respectively. The gray-shaded area around the regression line indicates the 95% confidence interval.

Point dendrometer data showed that growth was initiated in mid-April (day of year 135) and ended in mid-August (day of year 230) in 2009, with no systematic difference between treatments (defined as the day when 10% of the yearly growth increment was reached, Wilcoxon rank-sum test, *p* = 0.4). Stem water deficit derived from dendrometer readings did not differ systematically between the two groups (Figure 3A). Soil moisture under CO_2_-enriched trees tended to be higher than under control trees, but this difference was already present before the onset of the treatment in the first year and disappeared in the second year (Figure 3B). Decreasing mean daily soil moisture generally increased mean daily stem water deficit sharply, but as judged by the AIC the pattern did not change between the two groups, neither before nor after the start of the CO_2_-treatment (Figure 4). Stem storage saturation occurred at a wide spectrum of soil moisture contents, but depleted stem water reservoirs only occurred at low soil moisture values (<25 Vol. %, Figure 4).

**Figure 5:**
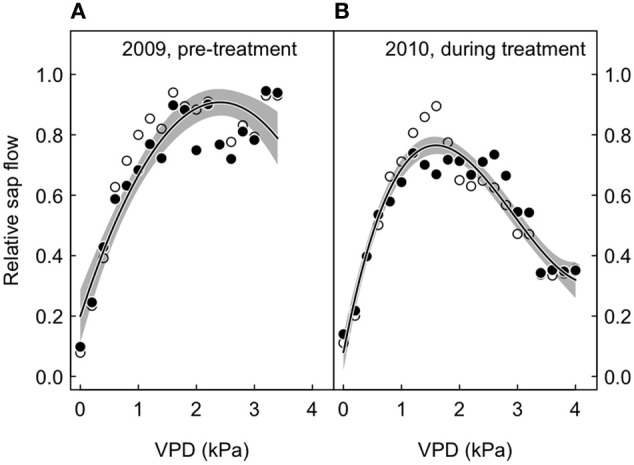
**Relative sap flow plotted against the vapor pressure deficit (VPD), according to treatment (open symbols, ambient; closed symbols, treated), before and after the initialization of the CO_2_-treatment.** Shown are 95 percentile values for 2 kPa bins, with non-linear fits (solid lines) and their 95% confidence intervals (gray-shaded area around the regression line). The merged model fits (treated and control trees) are shown. **(A)** 2009, before the start of the CO_2_ treatment. **(B)** 2010, during the CO_2_ treatment.

We also looked at the relative sap flow response to VPD according to experimental period (before and after treatment initiation). The 95th percentiles per 2 kPa bin did not differ between treatments, both before and after CO_2_ enrichment, because the interaction between VPD and CO_2_ in the polynomial model fits was statistically not significant (Figure 5).

### Comparison to modeling the CO_2_-response with LPJ-GUESS

Given the commonly implemented algorithms controlling leaf gas exchange in dynamic vegetation models, it can be expected that any PFT will show less water use under eCO_2_ (Farquhar et al., 1980). To see whether and to what extent this is the case in the commonly used DGVM LPJ-GUESS, we simulated our experiment with local climate data. We suppressed all other plant functional types but “needle-leaved evergreen.” The CO_2_ concentration for the 700-years spin-up was kept at 296 ppm. After 1901, the four CO_2_-scenarios outlined in the section “materials and methods” were imposed. The daily transpiration rates were sensitive to the final CO_2_ concentrations reached (550 ppm or 700 ppm), but only little to whether a step or gradual change in the CO_2_ concentration was used. Overall, there was c. 10% less transpiration in the 550 ppm scenario and c. 17% less transpiration in the 700 ppm scenario, regardless of how the concentration was reached or how long the new atmospheric conditions lasted (Table 1).

## Discussion

We aimed to assess the response of whole-tree water relations to future levels of atmospheric CO_2_ in Norway spruce, one of the most abundant and economically important coniferous tree species in Europe. Using five different, fully independent approaches (sap flow-, dendrometer-, leaf water potential-, and soil moisture measurements), our experimental data shows that this species is unlikely to adjust its water use under atmospheric CO_2_ concentrations anticipated to occur in c. 2050. This stands in contrast to the model results of the DGVM LPJ-GUESS, which predicts a c. 10% reduction in transpiration when simulating needle-leaved evergreen trees under CO_2_ concentrations and site conditions matching those of the field experiment. Because of the wide distribution across Europe and Asia and its importance as a timber species the lack of a water use response of Norway spruce to elevated atmospheric CO_2_ is fundamental for the parameterization and validation of DGVMs and fully coupled earth system models predicting the future water and carbon cycle. Further, it is central to our understanding of plant responses to eCO_2_ and how they are scaled with ontogeny and the successional stage of the tested species or community.

Critical to the estimates of net plant water use under eCO_2_ seem to be the developmental stage of the tested individuals, the species and PFT tested, the duration of the treatment, the method of CO_2_ administration, and the nature and timing of the measured traits used as a proxy to estimate plant water use (Table 2). All these factors will have an impact on the net response of plant water use to eCO_2_. Despite the large range of responses reported, the majority of studies tend to predict a decrease in stomatal conductance and therefore net water use under eCO_2_ (e.g., Curtis and Wang, 1998; Medlyn et al., 2001). However, there are numerous examples that show no response or even an increase in water use under eCO_2_. For *P. abies*, the species tested in the present study, Roberntz and Stockfors (1998) found no effect on *g*_*s*_ using branch bags, and Barton et al. (1993), Kupper et al. (2006) and Uddling et al. (2009) all report an increase in water use under eCO_2_ from glasshouse and branch bag experiments. For *Pinus taeda*, both a branch-bag and an open-top chamber experiment suggest that this species does not respond to eCO_2_ in its water use (Teskey, 1995; Pataki et al., 1998). Contrary to grassland experiments, Domec et al. (2009) found reduced stomatal conductance under eCO_2_ in tall, 28 years old *Pinus taeda* individuals only at high soil moisture, and no response during dry conditions.

**Table 2:** **Possible processes that contribute to the mitigation of the response of plant water use to eCO_2_**.

**Observed process/characteristic of experiment**	**Stomatal conductance (*g*_*s*_) or net water use/evapotranspiration per unit surface area**	**Example references**
Duration of the experiment	Decrease with duration of experiment	Medlyn et al., 2001; Leuzinger et al., 2011b
Developmental stage of study plants, canopy closure	Decrease with increasing maturity/canopy closure	Medlyn et al., 2001; Uddling et al., 2009
Plant functional type	Decrease from herbaceous to woody plants and from deciduous to coniferous trees	Saxe et al., 1998; Medlyn et al., 2001; Ainsworth and Rogers, 2007
Combination with other global change drivers	Trend for decrease	Leuzinger et al., 2011b
Scaling from plant to canopy/landscape	Decrease with increasing scale	Field et al., 1995; Wullschleger et al., 2002
Upscaling from experimental period to yearly average response	Responses-dependent on weather conditions, average water use often lower than if extrapolated linearly from experimental period	Wullschleger and Norby, 2001; Cech et al., 2003; Leuzinger and Körner, 2007
Soil feedback	Decrease (through wetter soils)	Schäfer et al., 2002

The circumstances that lead to this decline can generally be associated with a more realistic scenario (young vs. mature stands, local vs. global scale response etc.)

In trees that are in an early (expanding) successional stage, increased LAI under eCO_2_ may periodically (over-) compensate reductions in stomatal conductance (Li et al., 2003) or lead to an increase in total water use (Bobich et al., 2010). Increasing LAI following CO_2_-treatment has been reported for a closed-canopy *Pinus taeda* stand growing at the Duke experimental forest (McCarthy et al., 2007). However, this canopy response to eCO_2_ was determined by nitrogen availability patterns and additional N fertilization trials suggested that LAI stimulation is unlikely to occur at high fertility sites (McCarthy et al., 2007). Most other eCO_2_ studies suggest that CO_2_ will not cause an increase in LAI in mature systems (Bader et al., in preparation; Körner et al., 2005; Norby et al., 2005; Warren et al., 2011). Hättenschwiler and Körner (1997) even found a lower LAI under eCO_2_ in a young, closed-canopy Norway spruce stand, similar to what tropical tree model ecosystems revealed after stand closure (Körner and Arnone, 1992). Furthermore, a LAI beyond c. 2.7 will not affect canopy conductance (Schulze et al., 1994). Given the age of our study trees (>100 years) and the nutrient-rich soil they thrive on, it seems improbable that CO_2_ enrichment will enhance LAI in this stand.

We argue that the majority of the evidence underlying the existing reviews on plant water savings under eCO_2_ experiments (Curtis and Wang, 1998; Medlyn et al., 2001; Ainsworth and Long, 2005; Ainsworth and Rogers, 2007) rests on short-term (sub-seasonal) experiments, predominantly on grasslands, tree seedlings or juveniles tested under relatively confined conditions (e.g., glasshouses). However, these test conditions tend to overestimate the effects of eCO_2_ on stomatal conductance and/or water use and potential long-term alterations such as structural changes in the hydraulic pathway as have been reported for *Pinus taeda* at the Duke FACE site may remain undetected (Table 2; Domec et al., 2009).

Another source for overestimating water use under eCO_2_ is temporal upscaling from an experimental period shorter than or non-representative of a whole year (Leuzinger and Körner, 2007, 2010). Often, the response strongly depends on the weather and soil moisture conditions (Wullschleger and Norby, 2001; Cech et al., 2003). Our study, together with others (Ellsworth, 1999; Schäfer et al., 2002; Bernacchi et al., 2003; Keel et al., 2007; Uddling et al., 2009), supports the view that near-natural conditions tend to yield a smaller or no response in annual water use to elevated CO_2_.

Apart from the obvious absence of a CO_2_-response in the water flux of our experimental trees, the patterns found (Figure 2) match with earlier reports on water relations of *P. abies* (e.g., Gross and Koch, 1991; Zweifel et al., 2001). The low pre-dawn shoot water potentials around −1 MPa resulted from a combination of soil water deficits and the hydrostatic water potential of c. 0.4 MPa. The diurnal courses of water relations also showed tight stomatal control over transpiration, preventing midday shoot water potentials from dropping below −1.9 MPa. This rather isohydric behavior allowed the maintenance of an adequate hydraulic safety margin from the critical threshold of −2.5 MPa that has been reported as turgor loss point (Gross and Koch, 1991) and as the level at which significant xylem cavitation occurs in branches of adult Norway spruce trees (Cochard, 1992; Lu et al., 1995). Compared to the first day of measurements (Figure 2, left panels), stomatal conductance decreased due to lower soil moisture or higher VPD, causing considerably less sap flow (Figure 2, middle and right panels). The incomplete night-time recovery of stem radius during times of low soil moisture (<30 vol. %) and high VPD suggests that internal water storage tissues could not be replenished and is thus indicative of a tree water deficit (Zweifel et al., 2005 Figure 3). We have no explanation for the high pre-dawn stomatal conductance measured in the morning of July 14 (Figure 2).

The modeled reduction in water use by coniferous trees for this site stands in contrast to our *in situ* measurements. The LPJ-GUESS dynamic vegetation model consistently predicted between 9% and 18% reduced transpiration, with the ambient CO_2_ concentration (*C*_*a*_, 550 ppm vs. 700 ppm) causing most of the sensitivity of the response and the mode of increase (step vs. gradual) as well as the duration of the new conditions (immediately after the increase vs. 100 years later) being rather insignificant. In LPJ-GUESS, but also in other dynamic vegetation models, the first-order response originates from the photosynthesis model through stomatal closure due to increased intercellular CO_2_ concentrations (*C*_*i*_). Because *C*_*i*_/*C*_*a*_ is assumed constant, changes in *C*_*a*_ will result in proportional changes in stomatal conductance. However, in LPJ-GUESS this effect only manifests itself during ample water supply (see section “Materials and Methods”). Therefore, the CO_2_-response does not increase with dry conditions, which has been found earlier (Hickler et al., 2008), although a carry-over effect from wet to dry periods (higher available soil moisture under eCO_2_) is possible. This behavior certainly does not mirror observations from grassland (Niklaus et al., 1998; Morgan et al., 2004) and the drought × eCO_2_ interactions in trees do not seem to be uniform (Beerling et al., 1996; Heath, 1998; Cech et al., 2003; Leuzinger and Körner, 2007 and others).

The key difference between photosynthesis models that are employed in dynamic vegetation models is essentially the formulation of *C*_*i*_/*C*_*a*_ (Katul et al., 2000). Therefore, the range of modeled responses is relatively narrow (Luo et al., 2008) and foreseeable, unless model-specific feedback mechanisms dampen or enhance the initial signal. For example, increased LAI through altered carbon allocation patterns, soil moisture, or atmospheric feedback (in a fully coupled model) could all contribute to changing the initial response largely prescribed by the photosynthesis model. The fact that our modeled water relations response to eCO_2_ seems largely-independent of the time the eCO_2–conditions_ are in place suggests that very little feedback mechanisms contribute to altering the first-order response, which seems to be stable across models and ecosystem types (Luo et al., 2008). Another important component potentially responsible for mitigating the CO_2_-response are the leaf and canopy boundary layer resistances, which are in series with the stomatal resistance but not explicitly considered in LPJ-GUESS (McNaughton and Jarvis, 1991).

Clearly, there are limitations as to what conclusions can be drawn from a sample of five adult Norway spruce individuals treated with elevated atmospheric CO_2_ over two seasons only. Such experiments struggle with the inherent trade-off between sample size and the realism of the experimental setting. While it may be more satisfying to get statistically more robust results on CO_2_-responses with young trees, we make little progress if young trees respond differently to mature trees (Medlyn et al., 2001; Uddling et al., 2009; Leuzinger et al., 2011a,b). More data on water use under eCO_2_ are needed from large-scale studies in mature systems to confirm our results. Equally important is the continuation of large-scale experiments over many years in order to alleviate some of the statistical shortcomings from originating from low replication.

In conclusion, we find a contrasting response between our experimental results with mature *P. abies* trees, and the model output from the global dynamic vegetation model LPJ-GUESS. Our experimental results are corroborated by evidence from studies on other plant functional types, and we argue that the more realistic the testing conditions, the smaller the water savings in response to eCO_2_. The modeled water relations response to eCO_2_ was strikingly robust both in this and previous studies. Because the modeled responses are closer to the leaf-level than to the ecosystem response in experiments, one explanation for the apparent discrepancy is that the propagation of the response from the leaf to the ecosystem is not captured appropriately in the currently available models. Attempts should be made to account for such processes in models potentially mitigating first-order CO_2_-effects on plant water use.

### Conflict of interest statement

The authors declare that the research was conducted in the absence of any commercial or financial relationships that could be construed as a potential conflict of interest.

## References

[B1a] Ainsworth E. A. Long S. P. (2005). What have we learnt from 15 years of free-air CO_2_ enrichment (FACE)? A meta-analytic review of the responses of photosynthesis, canopy properties and plant production to rising CO_2_. Tansley Rev. New Phytol. 165, 351–372 10.1111/j.1469-8137.2004.01224.x 15720649

[B1] Ainsworth E. A. Rogers A. (2007). The response of photosynthesis and stomatal conductance to rising [CO_2_]: mechanisms and environmental interactions. Plant Cell Environ. 30, 258–270 10.1111/j.1365-3040.2007.01641.x 17263773

[B2] Barton C. V. M. Lee H. S. J. Jarvis P. G. (1993). A branch bag and CO_2_ control-system for long-term CO_2_ enrichment of mature Sitka spruce Picea-sitchensis (Bong) carr. Plant Cell Environ. 16, 1139–1148

[B3] Beerling D. J. Heath J. Woodward F. I. Mansfield T. A. (1996). Drought-CO_2_ interactions in trees: observations and mechanisms. New Phytol. 134, 235–242

[B3a] Bernacchi C. J. Calfapietra C. Davey P. A. Wittig V. E. Scarascia-Mugnozza G. E. Raines C. A. (2003). Photosynthesis and stomatal conductance responses of poplars to free-air CO_2_ enrichment (PopFACE) during the first growth cycle and immediately following coppice. New Phytol. 159, 609–621 10.1046/j.1469-8137.2003.00850.x 33873598

[B4] Bert D. Leavitt S. W. Dupouey J. L. (1997). Variations of wood delta C-13 and water-use efficiency of Abies alba during the last century. Ecology 78, 1588–1596

[B5] Betts R. A. Boucher O. Collins M. Cox P. M. Falloon P. D. Gedney N. (2007). Projected increase in continental runoff due to plant responses to increasing carbon dioxide. Nature 448, 1037–1041 10.1038/nature06045 17728755

[B6] Bobich E. G. Barron-Gafford G. A. Rascher K. G. Murthy R. (2010). Effects of drought and changes in vapour pressure deficit on water relations of Populus deltoides growing in ambient and elevated CO(2). Tree Physiol. 30, 866–875 10.1093/treephys/tpq036 20462939

[B7] Boucher O. Jones A. Betts R. A. (2009). Climate response to the physiological impact of carbon dioxide on plants in the Met Office Unified Model HadCM3. Clim. Dynam. 32, 237–249

[B8] Cech P. G. Pepin S. Körner C. (2003). Elevated CO_2_ reduces sap flux in mature deciduous forest trees. Oecologia 137, 258–268 10.1007/s00442-003-1348-7 12898382

[B8a] Cochard H. (1992). Vulnerability of several conifers to air embolism. Tree Physiol. 11, 73–83 10.1093/treephys/11.1.73 14969968

[B9] Curtis P. S. Wang X. Z. (1998). A meta-analysis of elevated CO_2_ effects on woody plant mass, form, and physiology. Oecologia 113, 299–313 10.1007/s004420050381 28307814

[B10] Domec J.-C. Palmroth S. Ward E. Maier C. A. Therezien M. Oren R. (2009). Acclimation of leaf hydraulic conductance and stomatal conductance of *Pinus taeda* (loblolly pine) to long-term growth in elevated CO(2) (free-air CO(2) enrichment) and N-fertilization. Plant Cell Environ. 32, 1500–1512 10.1111/j.1365-3040.2009.02014.x 19558405

[B11] Ellsworth D. S. (1999). CO_2_ enrichment in a maturing pine forest: are CO_2_ exchange and water status in the canopy affected? Plant Cell Environ. 22, 461–472

[B12] Farquhar G. D. Caemmerer S. V. Berry J. A. (1980). A biochemical-model of photosynthetic CO_2_ assimilation in leaves of C-3 species. Planta 149, 78–90 10.1007/BF00386231 24306196

[B13] Field C. B. Jackson R. B. Mooney H. A. (1995). Stomatal responses to increased CO_2_, implications from the plant to the global scale. Plant Cell Environ. 18, 1214–1225

[B14] Francey R. J. Farquhar G. D. (1982). An explanation of C-13/C-12 variations in tree rings. Nature 297, 28–31

[B15] Granier A. (1985). A new method of sap flow measurement in tree stems. Ann. Sci. For. 42, 193–200

[B16] Gross K. Koch W. (1991). Water relations of picea-abies.1. Comparison of water relations parameters of spruce shoots examined at the end of the vegetation period and in winter. Physiol. Plantarum 83, 290–295

[B17] Hartmann H. (2011). Will a 385 million year-struggle for light become a struggle for water and for carbon? – How trees may cope with more frequent climate change-type drought events. Glob. Change Biol. 17, 642–655

[B18] Hättenschwiler S. Körner C. (1997). Annual CO_2_ budget of spruce model ecosystems in the third year of exposure to elevated CO2. Acta Oecol. 18, 319–325

[B19] Haxeltine A. Prentice I. C. (1996). A general model for the light-use efficiency of primary production. Funct. Ecol. 10, 551–561

[B20] Heath J. (1998). Stomata of trees growing in CO_2_-enriched air show reduced sensitivity to vapour pressure deficit and drought. Plant Cell Environ. 21, 1077–1088

[B21] Hickler T. Smith B. Prentice I. C. Mjofors K. Miller P. Arneth A. (2008). CO_2_ fertilization in temperate FACE experiments not representative of boreal and tropical forests. Glob. Change Biol. 14, 1531–1542

[B22] Holtum J. A. M. Winter K. (2010). Elevated CO_2_ and forest vegetation: more a water issue than a carbon issue? Funct. Plant Biol. 37, 694–702

[B23] Housman D. C. Naumburg E. Huxman T. E. Charlet T. N. Nowak R. S. Smith S. D. (2006). Increases in desert shrub productivity under elevated carbon dioxide vary with water availability. Ecosystems 9, 374–385

[B24] Jarvis P. G. (1976). Interpretation of variations in leaf water potential and stomatal conductance found in canopies in field. Philos. Trans. R. Soc. Lond. B Biol. Sci. 273, 593–610 10.1098/rstb.2014.0311 PMC4360119 25750234

[B25] Katul G. G. Ellsworth D. S. Lai C. T. (2000). Modelling assimilation and intercellular CO_2_ from measured conductance: a synthesis of approaches. Plant Cell Environ. 23, 1313–1328

[B25a] Keel S. G. Pepin S. Leuzinger S. Körner C. (2007). Stomatal conductance in mature deciduous forest trees exposed to elevated CO_2_. Trees 21, 151–159 10.1126/science.1113977 16123297

[B26] Kimball B. A. Idso S. B. Johnson S. Rillig M. C. (2007). Seventeen years of carbon dioxide enrichment of sour orange trees: final results. Glob. Change Biol. 13, 2171–2183

[B26a] Körner C. Arnone J. A. 3rd. (1992). Responses to elevated carbon dioxide in artificial tropical ecosystems. Science 257, 1672–1675 10.1126/science.257.5077.1672 17841166

[B27] Körner C. (2006). Plant CO_2_ responses: an issue of definition, time and resource supply. New Phytol. 172, 393–411 10.1111/j.1469-8137.2006.01886.x 17083672

[B28] Körner C. (2009). Responses of humid tropical trees to rising CO(2). Annu. Rev. Ecol. Evol. Syst. 40, 61–79

[B29] Körner C. Asshoff R. Bignucolo O. Hattenschwiler S. Keel S. G. Pelaez-Riedl S. (2005). Carbon flux and growth in mature deciduous forest trees exposed to elevated CO_2_. Science 309, 1360–1362 10.1126/science.1113977 16123297

[B30] Körner C. Morgan J. Norby R. (2007). Terrestrial Ecosystems in a Changing World. CO_2_ Fertilization: When, Where, How Much? Berlin: Springer

[B31] Kupper P. Sellin A. Klimankova Z. Pokorny R. Puertolas J. (2006). Water relations in Norway spruce trees growing at ambient and elevated CO_2_ concentrations. Biol. Plantarum 50, 603–609

[B32] Leuning R. (1995). A critical-appraisal of a combined stomatal-photosynthesis model for C-3 plants. Plant Cell Environ. 18, 339–355

[B33] Leuzinger S. Hartmann A. Körner C. (2011a). Water relations of climbing ivy in a temperate forest. Planta 233, 1087–1096 10.1007/s00425-011-1363-6 21293876

[B35] Leuzinger S. Luo Y. Q. Beier C. Dieleman W. Vicca S. Körner C. (2011b). Do global change experiments overestimate impacts on terrestrial ecosystems? Trends Ecol. Evol. 26, 236–241 10.1016/j.tree.2011.02.011 21444122

[B34] Leuzinger S. Körner C. (2007). Water savings in mature deciduous forest trees under elevated CO_2_. Glob. Change Biol. 13, 2498–2508

[B34a] Leuzinger S. Körner C. (2010). Rainfall distribution is the main driver of runoff under future CO_2_-concentration in a temperate deciduous forest. Glob. Change Biol. 16, 246–254

[B36] Li J. H. Dugas W. A. Hymus G. J. Johnson D. P. Drake B. G. Hungate B. A. (2003). Direct and indirect effects of elevated CO_2_ on transpiration from Quercus myrtifolia in a scrub-oak ecosystem. Glob. Change Biol. 9, 96–105

[B37] Long C. Bala G. Caldeira K. Nemani R. Ban-Weiss G. (2010). Importance of carbon dioxide physiological forcing to future climate change. Trends Plant Sci. 15, 5–10 10.1073/pnas.0913000107 20445083 PMC2906877

[B37a] Lu B. Biron P. Bréda N. Granier A. (1995). Water relations of adult Norway spruce (Picea abies (L) Karst) under soil drought in the Vosges mountains: water potential, stomatal conductance and transpiration. Ann. For. Sci. 52, 117–129

[B38] Luo Y. Q. Gerten D. Le Maire G. Parton W. J. Weng E. S. Zhou X. H. (2008). Modeled interactive effects of precipitation, temperature, and [CO2] on ecosystem carbon and water dynamics in different climatic zones. Glob. Change Biol. 14, 1986–1999

[B38a] McCarthy H. R. Oren R. Finzi A. C. Ellsworth D. S. Kim H.-S. Johnsen K. H. (2007). Temporal dynamics and spatial variability in the enhancement of canopy leaf area under elevated atmospheric CO_2_. Glob. Change Biol. 13, 2479–2497

[B39] McCarthy H. R. Oren R. Johnsen K. H. Gallet-Budynek A. Pritchard S. G. Cook C. W. (2010). Re-assessment of plant carbon dynamics at the Duke free-air CO_2_ enrichment site: interactions of atmospheric CO_2_ with nitrogen and water availability over stand development. New Phytol. 185, 514–528 10.1111/j.1469-8137.2009.03078.x 19895671

[B40] McNaughton K. G. Jarvis P. G. (1991). Effects of spatial scale on stomatal control of transpiration. Agric. For. Meteorol. 54, 279–302

[B41] Medhurst J. Parsby J. Linder S. Wallin G. Ceschia E. Slaney M. (2006). A whole-tree chamber system for examining tree-level physiological responses of field-grown trees to environmental variation and climate change. Plant Cell Environ. 29, 1853–1869 10.1111/j.1365-3040.2006.01553.x 16913874

[B42] Medlyn B. E. Barton C. V. M. Broadmeadow M. S. J. Ceulemans R. De Angelis P. Forstreuter M. (2001). Stomatal conductance of forest species after long-term exposure to elevated CO_2_ concentration: a synthesis. New Phytol. 149, 247–264 10.1046/j.1469-8137.2001.00028.x 33874628

[B43] Miller-Rushing A. J. Primack R. B. Templer P. H. Rathbone S. Mukunda S. (2009). Long-term relationships among atmospheric CO(2), stomata, and intrinsic water use efficiency in individual trees. Am. J. Bot. 96, 1779–1786 10.3732/ajb.0800410 21622298

[B44] Moorcroft P. R. (2006). How close are we to a predictive science of the biosphere? Trends Ecol. Evol. 21, 400–407 10.1016/j.tree.2006.04.009 16815439

[B45] Morgan J. A. Pataki D. E. Gruenzweig J. M. Körner C. Niklaus P. A. Polley H. W. (2003). Grassland productivity responses to rising atmospheric carbon dioxide are driven primarily by water relations. Ecol. Soc. Am. Ann. Meet. Abstr. 88, 243

[B46] Morgan J. A. Pataki D. E. Körner C. Clark H. Del Grosso S. J. Grunzweig J. M. (2004). Water relations in grassland and desert ecosystems exposed to elevated atmospheric CO_2_. Oecologia 140, 11–25 10.1007/s00442-004-1550-2 15156395

[B47] Niklaus P. A. Spinnler D. Körner C. (1998). Soil moisture dynamics of calcareous grassland under elevated CO_2_. Oecologia 117, 201–208 10.1007/s004420050649 28308488

[B48] Norby R. J. DeLucia E. H. Gielen B. Calfapietra C. Giardina C. P. King J. S. (2005). Forest response to elevated CO_2_ is conserved across a broad range of productivity. Proc. Natl. Acad. Sci. U.S.A. 102, 18052–18056 10.1073/pnas.0509478102 16330779 PMC1312431

[B49] Norby R. J. Warren J. M. Iversen C. M. Medlyn B. E. McMurtrie R. E. (2010). CO(2) enhancement of forest productivity constrained by limited nitrogen availability. Proc. Natl. Acad. Sci. U.S.A. 107, 19368–19373 10.1073/pnas.1006463107 20974944 PMC2984154

[B50] Norby R. J. Wullschleger S. D. Gunderson C. A. Johnson D. W. Ceulemans R. (1999). Tree responses to rising CO_2_ in field experiments: implications for the future forest. Plant Cell Environ. 22, 683–714

[B51] Pataki D. E. Huxman T. E. Jordan D. N. Zitzer S. F. Coleman J. S. Smith S. D. (2000). Water use of two Mojave Desert shrubs under elevated CO_2_. Glob. Change Biol. 6, 889–897

[B52] Pataki D. E. Oren R. Phillips N. (1998). Responses of sap flux and stomatal conductance of *Pinus taeda* L. Trees to stepwise reductions in leaf area. J. Exp. Bot. 49, 871–878

[B53] Penuelas J. Canadell J. G. Ogaya R. (2011). Increased water-use efficiency during the 20th century did not translate into enhanced tree growth. Glob. Ecol. Biogeogr. 20, 597–608

[B54] Pepin S. Körner C. (2002). Web-FACE: a new canopy free-air CO_2_ enrichment system for tall trees in mature forests. Oecologia 133, 1–9 10.1007/s00442-002-1008-3 24599362

[B55] R Development Core Team. (2011). R: A Language and Environment for Statistical Computing. Vienna: R Foundation for Statistical Computing

[B56] Roberntz P. Stockfors J. (1998). Effects of elevated CO_2_ concentration and nutrition on net photosynthesis, stomatal conductance and needle respiration of field-grown Norway spruce trees. Tree Physiol. 18, 233–241 10.1093/treephys/18.4.233 12651377

[B56a] Saxe H. Ellsworth D. S. Heath J. (1998). Tree and forest functioning in an enriched CO_2_ atmosphere. Tansley Rev. New Phytol. 139, 395–436

[B57] Schäfer K. V. R. Oren R. Lai C. T. Katul G. G. (2002). Hydrologic balance in an intact temperate forest ecosystem under ambient and elevated atmospheric CO_2_ concentration. Glob. Change Biol. 8, 895–911

[B58] Schulze E. D. Kelliher F. M. Körner C. Lloyd J. Leuning R. (1994). Relationships among maximum stomatal conductance, ecosystem surface conductance, carbon assimilation rate, and plant nitrogen nutrition – a global ecology scaling exercise. Ann. Rev. Ecol. Syst. 25, 629

[B59] Sitch S. Smith B. Prentice I. C. Arneth A. Bondeau A. Cramer W. (2003). Evaluation of ecosystem dynamics, plant geography and terrestrial carbon cycling in the LPJ dynamic global vegetation model. Glob. Change Biol. 9, 161–185

[B60] Smith B. Prentice I. C. Sykes M. T. (2001). Representation of vegetation dynamics in the modelling of terrestrial ecosystems: comparing two contrasting approaches within European climate space. Glob. Ecol. Biogeogr. 10, 621–637

[B61] Tank A. M. G. K. Wijngaard J. B. Konnen G. P. Bohm R. Demaree G. Gocheva A. (2002). Daily dataset of 20th-century surface air temperature and precipitation series for the European Climate Assessment. Int. J. Climatol. 22, 1441–1453

[B62] Teskey R. O. (1995). A field-study of the effects of elevated CO_2_ on carbon assimilation, stomatal conductance and leaf and branch growth of *Pinus taeda* trees. Plant Cell Environ. 18, 565–573

[B63] Tricker P. J. Pecchiari M. Bunn S. M. Vaccari F. P. Peressotti A. Miglietta F. (2009). Water use of a bioenergy plantation increases in a future high CO(2) world. Biomass Bioenergy 33, 200–208

[B64] Uddling J. Teclaw R. M. Kubiske M. E. Pregitzer K. S. Ellsworth D. S. (2008). Sap flux in pure aspen and mixed aspen-birch forests exposed to elevated concentrations of carbon dioxide and ozone. Tree Physiol. 28, 1231–1243 10.1093/treephys/28.8.1231 18519254

[B65] Uddling J. Teclaw R. M. Pregitzer K. S. Ellsworth D. S. (2009). Leaf and canopy conductance in aspen and aspen-birch forests under free-air enrichment of carbon dioxide and ozone. Tree Physiol. 29, 1367–1380 10.1093/treephys/tpp070 19773339

[B66] Walthert L. Zimmermann S. Blaser P. Luster J. Lüscher P. (2004). Waldböden der Schweiz. Band 1. Grundlagen und Region Jura. Bern: Hep Verlag, 768

[B67] Warren J. M. Poetzelsberger E. Wullschleger S. D. Thornton P. E. Hasenauer H. Norby R. J. (2011). Ecohydrologic impact of reduced stomatal conductance in forests exposed to elevated CO(2). Ecohydrology 4, 196–210

[B68] Wullschleger S. D. Gunderson C. A. Hanson P. J. Wilson K. B. Norby R. J. (2002). Sensitivity of stomatal and canopy conductance to elevated CO_2_ concentrationinteracting variables and perspectives of scale. New Phytol. 153, 485–496 10.1046/j.0028-646X.2001.00333.x 33863220

[B69] Wullschleger S. D. Norby R. J. (2001). Sap velocity and canopy transpiration in a sweetgum stand exposed to free-air CO2 enrichment (FACE). New Phytol. 150, 489–498

[B70] Zweifel R. Item H. Häsler R. (2001). Link between diurnal stem radius changes and tree water relations. Tree Physiol. 21, 869–877 10.1093/treephys/21.12-13.869 11498334

[B71] Zweifel R. Zimmermann L. Newbery D. M. (2005). Modeling tree water deficit from microclimate: an approach to quantifying drought stress. Tree Physiol. 25, 147–156 10.1093/treephys/25.2.147 15574396

